# The Draft Genome Sequence of *Xanthomonas* sp. Strain Mitacek01 Expands the Pangenome of a Genus of Plant Pathogens

**DOI:** 10.1128/genomeA.01450-15

**Published:** 2015-12-10

**Authors:** M. B. Couger, Radwa A. Hanafy, Rachel M. Mitacek, Connie Budd, Donald P. French, Wouter D. Hoff, Mostafa S. Elshahed, Noha H. Youssef

**Affiliations:** a Department of Microbiology and Molecular Genetics, Oklahoma State University, Stillwater, Oklahoma, USA; b Department of Integrative Biology, Oklahoma State University, Stillwater, Oklahoma, USA

## Abstract

We report the draft genome sequence of *Xanthomonas* sp. strain Mitacek01, isolated from an indoor environment vending machine surface with frequent human use in Stillwater, Oklahoma, USA, as part of the Student-Initiated Microbial Discovery project. The genome has a total size of 3,617,426 bp and a contig *N*_50_ of 1,906,967 bp.

## GENOME ANNOUNCEMENT

*Xanthomonas* spp. infect over 300 host plants, including many important crops such as rice (1). Such infections can result in reduction in crop yields or outright crop failures and hence could have a major impact on economic development and global food supply (2). Genomic analysis of strains belonging to the genus *Xanthomonas* can contribute to understanding the molecular mechanisms of pathogenesis and subsequently reduce the occurrence and/or mitigate the severity of such infections (3, 4).

*Xanthomonas* sp. strain Mitacek01 was isolated from an indoor environment vending machine surface and was sequenced on the Illumina MiSeq platform at the University of Georgia Genomics Facility using 2 × 300 paired-end chemistry. Generated reads were quality filtered with standard Illumina filtering settings resulting in 1,511,702 (453.5 Mb) quality sequences. All quality-filtered reads were assembled using the short read de Brujin graph assembly (5) program Velvet (6). Velvet assembly run-time settings used were a *k*-mer value of 101 bp and a minimum contig coverage value of 7×. Gene models were created using the prokaryotic gene calling software package Prodigal (7). The Velvet assembly had a total size of 3,617,426 bp and an *N*_50_ of 1,906,967 bp. The largest assembled contig was 1,906,967 bp, with a GC content of 68.5%. A total of 3,212 gene models were predicted. Translated protein sequences were functionally annotated using a combination of NCBI BLAST C++ homology search (8) and HMMER version 3.0 hmmscan (9) against the PFAM 26.0 database (10).

16S rRNA gene-based comparisons to *Xanthomonas* genomes publicly available in the GenBank database (*n* = 302,955,543, October 2015) revealed that strain Mitacek01 was closely related (97.0% similarity) to *Xanthomonas oryzae* pv. oryzicola strain YM15, a causative agent of bacterial leaf streak in rice (11), *Xanthomonas campestris* strain 17, a phytopathogen capable of infecting a wide range of plants (12), and 21 different strains of *Xanthomonas citri*, causative agents of citrus canker (13). Despite close 16S rRNA gene sequence similarity to multiple *Xanthomonas* strains, BLAST analysis identified 393 genes (12.2%) within the Mitacek01 genome with no sequence homology (e value <10^−05^) to any of the genes in the *Xanthomonas* pan genome (genomes = 32; protein sequences = 298,975; October 2015). The majority of these genes were hypothetical (147/393, 37.4%) and conserved hypothetical (186/393, 47.3%) proteins. In addition, genes encoding β-lactamase, glucolactone synthesis, and phenol metabolism were identified, further expanding the pan-metabolic repertoire of the genus *Xanthomonas*.

To identify putative virulence factors we used signalP (14) to identify secreted proteins and compared gene models to the pathogen-host interactions (PHI) (15) database to identify genes that had been previously established to affect *Xanthomonas* pathogenesis. A total of 497 secreted genes were identified, and 29 genes in the genome were present in the PHI database.

In conclusion, this initial genomic analysis of strain Mitacek01 highlights a high level of intergenomic diversity within the genus *Xanthomonas*, supporting previous findings for this genus (16, 17) and contributing to the available genomic resources for the study of an economically relevant group of phytopathogens.

### Nucleotide sequence accession number.

The draft genome of *Xanthomonas* sp. strain Mitacek01 has been deposited in GenBank under the accession number LKIT00000000.
